# Development of an innovative, competency-based, multi-speciality training programme in global health for graduate medical education

**DOI:** 10.7189/jogh.15.03029

**Published:** 2025-08-04

**Authors:** Elizabeth S Rose, Rondi M Kauffmann, Marie H Martin, J Matthew Kynes, Lipika Narisetti, Ryan H Belcher, Christopher M Bonfield, Kristen B Dettorre, Michael C Dewan, Joseline Haizel-Cobbina, Merranda D Holmes, Tolulope O Kehinde, Jonathan A Niconchuk, Kush Chaudhari, Annesa Dey, Jada S Sims, Teresa Y Xu, Lindsey E Zamora

**Affiliations:** 1 Institute for Global Health, Vanderbilt University Medical Center, Nashville, USA; 2 Department of Pediatrics, Vanderbilt University Medical Center, Nashville, USA; 3 Department of Surgery, Vanderbilt University Medical Center, Nashville, USA; 4 Department of Health Policy, Vanderbilt University Medical Center, Nashville, USA; 5 Department of Anesthesiology, Vanderbilt University Medical Center, Nashville, USA; 6 Vanderbilt University, Nashville, USA; 7 Department of Otolaryngology, Vanderbilt University Medical Center, Nashville, USA; 8 Department of Neurological Surgery, Vanderbilt University Medical Center, Nashville, USA; 9 Department of Emergency Medicine, Vanderbilt University Medical Center, Nashville, USA; 10 Department of Medicine, Vanderbilt University Medical Center, Nashville, USA; 11 Department of Obstetrics and Gynecology, Vanderbilt University Medical Center, Nashville, USA

## Abstract

Physicians increasingly care for ethnically diverse populations. Demand for global health training in graduate medical education (GME) is increasing, but there is variability across specialities. We developed a multi-speciality GME course for trainees seeking to integrate global health into their careers. The Consortium of Universities in Global Health’s Toolkit was the foundation for creating this course. The course includes eleven modules taught by faculty across specialities. Trainees completed pre- and post-course online surveys to measure their knowledge, skills, and attitudes on these global health competencies and to gather course feedback. We compared pre- and post-course data to determine change in global health competencies and course perceptions. In the first two years, 62 residents participated. Overall, trainees’ knowledge of international health concepts increased by 51%. The course was well received; 95% would recommend the course to a colleague. There was no fee to participate. Faculty donated their effort to developing and teaching the course. Faculty worked in pairs to maintain feasibility of implementation and were responsible for one module. Analyses showed the course had high levels of effectiveness in increasing learners’ knowledge, acceptability by learners, and feasibility of implementation. This programme collaborates across specialities and is a model for other GME programmes.

As our world becomes increasingly globalised, the international flow of people, products, and services has a substantial role in global health systems and health care delivery [1]. In 2020, there were an estimated 280 million international migrants worldwide. Within the USA, 14.3% of people are foreign-born [2]. This global migration has contributed to novel infectious disease transmission patterns and a rise in drug-resistant bacteria and microorganisms [3]. These challenges underscore the need for global health training to enhance physicians’ expertise in diagnosing and treating diverse pathologies while increasing their understanding of global health systems, sociocultural health disparities, and cross-cultural sensitivity.

Concurrent with this need, interest in global health training in graduate medical education (GME) or residency programmes is growing [4,5]. Participation in global health opportunities has positive effects, including improved confidence in providing culturally sensitive care to diverse populations, increased likelihood of practising in underserved or rural areas, and higher milestone achievement in the Accreditation Council for Graduate Medical Education competencies [6–8]. USA residency programmes are increasingly incorporating global health training through international rotations. Still, the availability of these opportunities varies amongst specialities, and rotations may be cost-prohibitive to residents, decreasing participation [9,10]. Additionally, many programmes face challenges in developing standardised, competency-based global health curricula to accompany and enhance the learning of residents participating in global health opportunities.

To address these challenges, we developed a multi-speciality year-long global health GME classroom-based course to train residents in foundational concepts in ethical, mindful engagement in global health partnerships. To our knowledge, this course is the first to train residents of all specialities in an integrated setting. We aimed to evaluate the course’s development, implementation, and outcomes.

## COURSE DEVELOPMENT

Recognising the multi-disciplinary nature of global health, we assembled faculty leaders of residency programmes from multiple specialities, including anaesthesiology, emergency medicine, general surgery, internal medicine, neurosurgery, otolaryngology, and obstetrics/gynaecology, who work at our institution and have expertise in global health. We used the Consortium of Universities in Global Health’s Global Health Education Competencies Toolkit to identify eleven core competencies foundational to a global health career, informing the course structure and resources [11]. Faculty dyads reviewed the toolkit and selected the competency domain in which they had the greatest expertise. Then, dyads reviewed the toolkit’s learning objectives and resources for their competency and selected objectives appropriate for medical residents in an introductory course and resources (*e.g.* articles, books, videos) that would help teach those objectives. Dyads incorporated outside resources as needed.

In designing the curriculum structure, we used a flipped-classroom model in which residents completed about two hours of asynchronous independent study (*e.g.* reading articles and watching videos on their own) before participating in monthly one-hour synchronous virtual classes. Each class covered a different competency and was taught by a faculty dyad. An outline of the classes, including learning objectives, materials, and discussion questions, is presented in the syllabus (Table S1 in the **Online Supplementary Document**). The faculty dyad expanded upon the study materials during classes and engaged residents in discussions. Several faculty members invited one of their international or local community partners as a co-presenter for the class. These guest presenters helped contextualise the class topic by sharing local perspectives and case studies related to the learning objectives. After class, the discussion continued in an online forum hosted on Microsoft Teams. The presenting faculty posted a question or case study related to the class topic, and residents responded to the questions and each other. Participation was high immediately after the class and then decreased throughout the month as residents moved on to focus on the next class topic.

## EVALUATION

We created a pre-course online survey using REDCap [12,13] to collect demographic data and baseline knowledge, skills, and attitudes (KSAs) related to course competencies. After the last class, we administered a post-course survey to measure changes in KSAs and gather feedback on course acceptability and feasibility. In both surveys, we asked participants to respond using five-point Likert-type scales and open-ended questions.

We calculated descriptive statistics of the quantitative data, including mean (x̄), standard deviation (SD), and percentage change between average pre- and post-course KSAs. We used Microsoft Excel’s AVERAGE and STDEV formulas to calculate x̄ and SD. To calculate the percent change for each KSA, we found the pre-course x̄ and post-course x̄; then, we calculated the difference between the post-course x̄ and the pre-course x̄, divided that number by the pre-course x̄, and multiplied by 100, or: (post-course x̄ − pre-course x̄) / pre-course x̄ × 100.

We used an inductive, semantic approach for the thematic analysis of qualitative responses to the open-ended survey questions. Two authors (RMK, MHM), with experience in qualitative analysis, independently coded qualitative responses and then identified key themes that arose from the codes. Two other authors (ESR, LEZ), also with qualitative analysis experience, reviewed these codes, and the codes were refined through group consensus, in which there was discussion until agreement. We used Microsoft Excel, version 16.97.2, to calculate all descriptive statistics and organise all qualitative responses.

## COURSE OUTCOMES

In the pilot years of this course, 62 residents representing 11 specialities enrolled and 59 (95%) completed the pre-course survey. About one-third (n = 19; 32%) completed the course, which is defined as active participation in at least 80% of the classes. Of those, 100% (n = 19) completed the post-course survey. In the post-course survey, residents cited scheduling conflicts, such as late clinics or overnight shifts, as the primary reasons for missing classes and not completing the course. While this course was intended for first-year residents, almost half (n = 9, 47%) completed it in their second year. Four of those graduates began the course in their first year but could not complete it, and carried over credits of completed classes from their first to second year. While surgery (n = 13; 23%) and internal medicine (n = 10; 18%) represented two of the most common specialities among participants, only 23% of surgery and 50% of internal medicine participants completed the course. All the otolaryngology participants (n = 2) completed the course. Paediatric (67%) and med-peds participants (60%) were the next likely to complete the course.

Among the 59 pre-course survey respondents, the most common prior global health experiences included volunteering (n = 37; 63%), leisure travel (n = 34; 58%), and study abroad (n = 24; 41%) (Table S2 in the **Online Supplementary Document**). Notably, 16 (27%) were born or raised abroad. Over one-third (n = 21; 36%) had prior global health training. Respondents desired to incorporate global health into their career, including conducting projects abroad (n = 44; 75%) and working with immigrants locally (n = 38; 64%).

On average, residents’ knowledge of course competencies increased 51% from before to after the course. Top growth areas included global availability of health care workers (x̄ = 71%), discipline-specific global health approaches (x̄ = 67%), and global health programme management (x̄ = 62%) (Table S3 in the **Online Supplementary Document**). The smallest increase occurred in social drivers of health (x̄ = 31%). However, residents reported the highest pre-course knowledge in this area, leaving less room for improvement.

Post-course survey results showed high confidence in course-related skills, particularly in identifying social drivers of health (x̄ = 4.11; SD = 0.66), global health ethics (x̄ = 4.16; SD = 0.69), and discipline-specific global health approaches (x̄ = 4.11; SD = 0.74) (Table S4 in the **Online Supplementary Document**). The average residents’ overall confidence in their course-related skills was 3.94 (SD = 0.15).

All residents who completed the post-course survey agreed that the course met their expectations, presented course content clearly, and allocated sufficient time for each class. All but one (95%) said they would recommend the course to a colleague. Most (n = 15, 79%) found the required independent study materials useful.

Residents described that the most helpful elements of the course were connecting across specialities and learning from guest lecturers (Table S5 in the **Online Supplementary Document**). Respondents elaborated on how connecting with peers across specialities enriched their experience. One respondent noted:

Training with residents from other specialities enriches the educational experience, enhances problem-solving skills, improves communication and teamwork, expands knowledge, and ultimately leads to improved patient care.

Additionally, residents emphasised that perspectives and real-life experiences from international partners provided context and increased their understanding of the topics. One wrote that the ‘most impactful lectures from the series were those that were presented directly from community partners’. Another noted, ‘I gained perspective from individuals in a variety of global health settings and hearing how they approach, think about, and overcome global health challenges’.

Most residents felt the course encompassed a broad range of topics, but some suggested teaching more practical skills, such as preparing for a clinical rotation, rather than focusing on theoretical discussions. They also expressed interest in learning about neglected tropical diseases, mental health, and careers in global health research. Residents suggested including ethics case discussions, real-world examples, and clinical ‘pearls’. They also desired more connections with faculty engaged in global health work.

Although residents felt the virtual format might limit discussions compared to an in-person format, they appreciated how it allowed for increased attendance and connecting between sessions. They suggested including an in-person orientation and a simulation laboratory session. Residents identified a busy residency schedule and clinical care responsibilities as barriers to attending classes.

## IMPLICATIONS

This global health course was well-received by a multi-speciality group of medical residents and provides a framework for teaching global health-related competencies in GME programmes. Among residents, 31% had prior global health training, and 5% reported no prior experience, with wide variability in prior experience working and travelling abroad. Despite this diversity, the course provided important training for residents of all backgrounds. Furthermore, this variety of experiences enriched group discussions, allowing residents to learn from each other.

In their written feedback, respondents overwhelmingly felt that the collaboration across specialities was one of the strongest aspects of this curriculum. Residency programmes and clinical practice in the USA is often conducted in speciality ‘silos’, and residents noted many ways that they appreciated connecting outside these silos, as it enhanced their global health community and understanding of other specialities. We feel that this teaching model – a multi-speciality community of practice – provides residents with a foundational community in global health where they can continue collaborating beyond the course. The cross-speciality nature of this course is important in fostering interdisciplinary communication and problem solving [14], which supports better-functioning teams and health systems domestically and abroad that ultimately improve patient care and outcomes.

In this programme’s two-year pilot, we opened the course to specialities where a faculty member was involved in developing and teaching the course. We believe this connection provided buy-in and support for the programme within specific specialities. Now that the programme is established, we plan to expand the course to other specialities and encourage new faculty to contribute to the course. However, although the course teaches across the specialities, one of the largest reported areas of knowledge and skills growth was understanding approaches for the specific discipline of the global health practitioner. This finding demonstrated that residents can still learn speciality specifics within a multi-speciality course with faculty from multiple disciplines.

The residents’ area of least growth was the social drivers of health. This low growth could be due to the high baseline knowledge in that competency, leaving less room for growth (*i.e.* ceiling effect). This knowledge area is not exclusive to global health, suggesting that residents may be more familiar with domestic health equity issues. However, this course included discussions about how social drivers of health manifest differently across regions and cultures, expanding upon residents’ prior knowledge of domestic matters and encouraging them to apply those concepts to contexts with international populations. Our findings may indicate a knowledge gap in global health-related topics and highlight the importance of including global health courses in mainstream GME programmes. While our residents had little increase in the knowledge of social drivers of health, a review of global health programmes found that global health training can increase residents’ understanding of social drivers of health [14].

Recognising that health equity challenges exist in the USA and abroad, it was encouraging that 65% of residents plan to work with underserved communities in the USA. Novel strategies are needed to address health care provider shortages in the USA’s rural and urban areas of low socioeconomic status. Including global health training in GME programmes is one strategy to increase residents’ awareness of health equity issues and encourage working in underserved areas.

The associated cost is one barrier for many learners to participate in global health international rotations. There was no cost to residents to participate in this programme, which we feel was an innovative approach to provide accessibility to global health skills training to all residents. This addresses a challenge met by traditional global health training programmes that focus solely on international rotations to teach global health competencies. In this classroom-based framework, residents can gain global health competencies, strengthen cross-speciality communication, and increase awareness of health equity issues, without costly international rotations. However, we acknowledge that some residents may have been deterred from participating due to the conventional mindset that global health learning can only be achieved through international travel, not through courses such as this or work with diverse domestic populations.

Only 32% of enrolled residents in these two pilot cohorts finished the course. Scheduling conflicts were the most cited reason for not completing the course, and we noted that residents in the specialities most likely to complete the course (*e.g.* paediatrics and med-peds) tend to have more consistent schedules and availability to meet after ‘normal’ working hours, thus increasing their ability to participate regularly in classes. Our findings align with Hau and colleague’s [10] findings in their systematic review of global health residency training programmes that medical specialities face fewer challenges to participating in training than surgical specialities. To improve the course completion rate, we moved the class start time two hours later (from 05:00 pm to 07:00 pm) for the third cohort, which is ongoing, and we have noticed increased attendance. Additionally, we allowed residents to ‘make up’ a session by watching the class recording and writing a one-page reflection on the lessons they learned related to the class topic. Several residency programmes at our institution have now included course completion as a requirement of their programme’s global health track and to earn the GME Global Health Certificate. We found that programmes that included this course in their programme’s global health track had a greater rate of course completion, which we believe could be an important factor of the course.

Residents also noted in their feedback that the course could be strengthened with more clinical ‘pearls’, ethics case discussions, and taught practical skills. We have incorporated some of these suggestions, such as including more ethics case discussions, into course revisions. To help clarify expectations for this course, we have included in the course orientation more details about the framework of this course as a broad introductory course that lays a foundation for ethical, mindful engagement with global health partners, noting that residents will have opportunities for more specific, clinical skills and knowledge in their speciality programme following completion of the course.

In creating this course, we did not receive institutional funding for faculty time in developing or teaching the course. We used institutional technological resources, including Microsoft Teams and SharePoint. This course can be replicated and implemented only with significant faculty buy-in and leadership. Before this course, our institution had robust global health programmes in some specialities, but lacked such programmes in others, particularly those with smaller numbers of residents and faculty. We found it beneficial to collaborate in creating a cross-speciality programme so as to leverage faculty enthusiasm and time in the smaller programmes. This model was feasible in an institution with faculty enthusiasm for working together across specialities.

Our study was limited to self-reported data from a small sample size collected immediately after completing the course. Hence, the evaluation of this pilot training is subject to several biases. To address these biases in future studies, observations and reports of residents engaging in global health activities would provide more insight into the ways they demonstrate skills in the course competencies, and longitudinal data, which we plan to gather in the coming years, will provide insight into the long-term impacts of this course. As we expand this course, we will have larger sample sizes, which will provide additional data points and information about the potential effects of this course. This study lacked a control group, which would have helped determine whether any of these competencies were strengthened outside the course. Finally, our international collaborators were mostly featured through co-presentation. As we continue to develop this course, we hope to involve them more heavily in module design so their knowledge can be more centred.

This global health GME course was developed and taught by faculty from multiple specialities. Broad faculty enthusiasm in creating this course, the cross-speciality nature of the course, and contributions from international guest lecturers were key strengths. Although residents had a range of global health experiences, the course helped all residents strengthen their knowledge and skills in eleven global health competencies and build community. With appropriate dedication and energy, this course model can be scaled and used as a framework for global health GME programmes.

## Additional material


Online Supplementary Document

## References

[1] Harrison R Meyer L Chauhan A Agaliotis M What qualities are required for globally-relevant health service managers? An exploratory analysis of health systems internationally. Global Health. 2019;15:11. 10.1186/s12992-019-0452-3 30728049 PMC6364385

[2] United States Census Bureau. Populations and People: Native and Foreign-born. 2023. Available: https://data.census.gov/profile/United_States?g=010XX00US. Accessed: 29 May 2025.

[3] Baker RE Mahmud AS Miller IF Rajeev M Rasambainarivo F Rice BL Infectious disease in an era of global change. Nat Rev Microbiol. 2022;20:193–205. 10.1038/s41579-021-00639-z 34646006 PMC8513385

[4] Reece J Dionne C Krupica T Lerfald N Sizemore J Sofka S Can global health opportunities lead to an increase in primary care physicians? J Glob Health. 2020;10:020387. 10.7189/jogh.10.020387 33282217 PMC7688187

[5] Kaeppler C Holmberg P Tam RP Porada K Stryker SD Conway K The impact of global health opportunities on residency selection. BMC Med Educ. 2021;21:384. 10.1186/s12909-021-02795-5 34266446 PMC8280583

[6] Stryker SD Conway K Kaeppler C Porada K Tam RP Holmberg PJ Underprepared: influences of U.S. medical students’ self-assessed confidence in immigrant and refugee health care. Med Educ Online. 2023;28:2161117. 10.1080/10872981.2022.2161117 36594616 PMC9815430

[7] Liaw W Bazemore A Xierali I Walden J Diller P Impact of Global Health Experiences During Residency on Graduate Practice Location: A Multisite Cohort Study. J Grad Med Educ. 2014;6:451–6. 10.4300/JGME-D-13-00352.1 26279768 PMC4535207

[8] Hayward AS Lee SS Douglass K Jacquet GA Hudspeth J Walrath J The Impact of Global Health Experiences on the Emergency Medicine Residency Milestones. J Med Educ Curric Dev. 2022;9:23821205221083755. 10.1177/23821205221083755 35572845 PMC9102119

[9] Kerry VB Walensky RP Tsai AC Bergmark RW Bergmark BA Rouse C US medical specialty global health training and the global burden of disease. J Glob Health. 2013;3:020406. 10.7189/jogh.03.020406 24363924 PMC3868823

[10] Hau DK Smart LR DiPace JI Peck RN Global health training among US residency specialties: a systematic literature review. Med Educ Online. 2017;22:1270020. 10.1080/10872981.2016.1270020 28178918 PMC5328369

[11] Consortium of Universities for Global Health. Global Health Competencies Toolkit. 2018. Available: https://www.cugh.org/online-tools/competencies-toolkit/. Accessed: 6 June 2021.

[12] Harris PA Taylor R Thielke R Payne J Gonzalez N Conde JG Research electronic data capture (REDCap)—a metadata-driven methodology and workflow process for providing translational research informatics support. J Biomed Inform. 2009;42:377–81. 10.1016/j.jbi.2008.08.010 18929686 PMC2700030

[13] Harris PA Taylor R Minor BL Elliott V Fernandez M O’Neal L The REDCap consortium Building an international community of software platform partners. J Biomed Inform. 2019;95:103208. 10.1016/j.jbi.2019.103208 31078660 PMC7254481

[14] Coria AL Rabin TL Rule AR Haq H Hudspeth JC Ratner L Global health crisis, global health response: how global health experiences prepared North American physicians for the COVID-19 pandemic. J Gen Intern Med. 2022;37:217–21. 10.1007/s11606-021-07120-w 34561829 PMC8475882

