# Supplementary material for: Development of an innovative, competency-based, multi-speciality training programme in global health for graduate medical education
Source: J Glob Health. 2025 Aug 4;15:03029. doi: 10.7189/jogh.15.03029 (PMC12319348; doi:10.7189/jogh.15.03029)
Supplement: Online Supplementary Document [file jogh-15-03029-s001.pdf]

Supplement to: Rose ES, Kauffmann RM, Martin MH, Kynes JM, Narisetti L, Belcher RH, Bonfield CM, Dettorre KB, Dewan MC, Haizel-Cobbina J, Holmes MD, Kehinde TO, Niconchuk JA, Chaudhari K, Dey A, Sims JS, Xu TY, Zamora LE. Development of an innovative, competency-based, multi-speciality training programme in global health equity for graduate medical education. J Glob Health. 2025;15:03029.

1. Table S1 Course syllabus (pages 2 -24)
2. Table S2 (pages 25-26)
3. Table S3 (page 27)
4. Table S4 (page 28)
5. Table S5 (page 29)

Table S1 Syllabus

VANDERBILT COLLABORATIVE FOR GLOBAL HEALTH EXCELLENCE (VCGHE)
SYLLABUS

COURSE OVERVIEW

Description
The Vanderbilt Collaborative for Global Health Excellence (VCGHE) exists to provide high-level training for graduate medical trainees seeking to integrate global health service, research, education, and development into their professional careers.

Aim/Purpose
VCGHE aims to use a combination of multidisciplinary and specialty-specific didactic teaching, small group discussion, mentorship, research, and practical clinical experiences to build the knowledge, skills and attitudes expected of physicians competent in global health work. These activities will be accomplished through a curriculum shared among all programs with monthly modules covering global health foundations for resident participants in their first year followed by specialty-specific education for residents in the remainder of the program.

Course Competencies (adapted from the *Consortium of Universities for Global Health*):

- Examine major causes of morbidity and mortality and their variations between high-, middle- and low-income regions, and with major public health efforts to reduce gaps in health outcomes globally.
- Understand how globalization affects health, health systems, and the delivery of health care.
- Identify social, economic, and environmental factors that are important contributors of health, and understand that health is more than the absence of disease.
- Explain the concept of capacity strengthening as the sharing of knowledge, skills, and resources for enhancing global public health programs, infrastructure, and workforce to address current and future global public health needs.
- Understand that genuine collaboration and partnership entails selecting, recruiting, and working with a range of global health stakeholders to advance research, policy, and practice goals, and to foster open dialogue and effective communication with partners and within a team.
- Apply basic principles of ethics to global health issues and settings.
- Identify approaches necessary for the specific profession or discipline of the global health practitioner.
- Utilize frameworks to address health differences across socially, demographically, or geographically defined populations.
- Explain how program management supports the design, implementation, and evaluation of global health programs to maximize contributions to effective policy, enhanced practice, and improved and sustainable health outcomes.
- Utilize sociocultural and political awareness to work effectively within diverse cultural settings and across local, regional, national, and international political landscapes.

Course Expectations

- Participants are expected to attend and participate in at least 80% of sessions (9 sessions)
- Class preparation includes completing reading assignments, which should take no more than 2 hours monthly
- Small groups are encouraged to engage in discussion and meet between monthly sessions
- Residents enrolled in the Global Health Excellence Availability and Leadership in Surgery certificate program are required to complete this course as part of their training

Meeting Dates and Times

- Meeting day/time: second Thursday of the month from 7:00-8:00pm on Teams

Monthly Module Topics and Dates

1. Introduction to Global Health and the Global Burden of Disease
2. Sociocultural Awareness in Global Health Engagements
3. Capacity Strengthening
4. Social & Environmental Considerations of Health and Planetary Health
5. Globalization of Health & Healthcare
6. Immigrant & Refugee Health
7. Collaboration, Partnering & Communication
8. Infections & Effects
9. Surgery, Anesthesia and Obstetrics
10. Program Management
11. Ethics & Neocolonialism

Making up sessions

If you miss a session, you may watch the recording. After watching the session, write a reflection on the session describing how the session deepened your global health knowledge/perspectives and how you might apply what you learned in the session to your clinical practice. Reflections should be at least one paragraph but do not need to exceed one page. Please email your reflection to the program directors. In general, you may submit reflections and receive "credit" for up to two sessions.

Program leaders

- Rondi Kauffmann, MD, MPH, FACS, FCS (ECSA) (rondi.kauffmann@vumc.org) Division of Surgical Oncology and Endocrine Surgery, Department of General Surgery
- Lindsey Zamora, MD, MPH (lindsey.zamora@vumc.org*)* Global Women's Health Section, Department of Obstetrics and Gynecology

Program faculty

- Ryan Belcher, MD, MPH (ryan.belcher@vumc.org) Department of Otolaryngology – Head and Neck Surgery
- Christopher Bonfield, MD (chris.bonfield@vumc.org) Division of Neurologic Surgery
- Kristen Dettorre, MD, DTMH (kristen.dettorre@vumc.org) Department of Emergency Medicine
- Michael Dewan, MD, MSCI (michael.dewan@vumc.org) Department of Neurologic Surgery
- Joseline Haizel-Cobbina, MBChB, MPH (joseline.haizel-cobbina@vumc.org), Global Neurosurgery Program
- Merranda Holmes, MD (merranda.d.holmes@vumc.org) Department of Medicine

- Rondi Kauffmann, MD, MPH, FACS, FCS (ECSA) (rondi.kauffmann@vumc.org) Division of Surgical Oncology and Endocrine Surgery, Department of Surgery
- Matthew Kynes, MD (j.matt.kynes@vumc.org) Department of Anesthesiology
- Marie Martin, PhD, MEd (marie.h.martin@vumc.org) Vanderbilt Institute for Global Health
- Jon Niconchuk, MD (Jonathan.a.niconchuk@vumc.org) Department of Anesthesiology
- Elizabeth Rose, EdD, MPH (elizabeth.rose@vumc.org) Vanderbilt Institute for Global Health
- Lindsey Zamora, MD, MPH (lindsey.zamora@vumc.org) Global Women's Health Section, Department of Obstetrics and Gynecology

Textbook and Primary Resources

- "Reimagining Global Health: An Introduction" by Paul Farmer, Jim Yong Kim, Arthur Kleinman, Matthew Basilico. University of California Press, 2013. https://www.amazon.com/Reimagining-Global-Health-Introduction-Anthropology/dp/0520271998
- Consortium of Universities for Global Health (CUGH) Competency Sub-Committee (2018). CUGH Global Health Education Competencies Tool Kit (2nd edition), Washington, DC.

MODULE 1. INTRODUCTION TO GLOBAL HEALTH AND THE GLOBAL BURDEN OF DISEASE

Learning Objectives

- Define global health
- Describe the major causes of morbidity and mortality around the world
- Become familiar with various metrics used to describe healthcare systems and their ability to meet the needs of the community

Milestones

CUGH Global Health Competencies 1a, 1b
ACGME Milestones:

- Systems-based practice
- Practice-based learning and improvement

Pre-reading and viewing list

- The Sustainable Development Goals: https://sdgs.un.org/2030agenda
- Fried LP, Bentley ME, Buekins P, Burke DS, et al. Global Health is Public Health. Lancet 2010 Feb 13;375(9714): 535-7. https://www.sciencedirect.com/science/article/pii/S0140673610602036?via%3Dihub
- Koplan JP, Bond TC, Merson MH, Reddy KS, Rodriguez MH, Sewankambo NK, and Wasserheit JN. Towards a common definition of global health. Lancet 2009 June 6; 373(9670):1993-1995.
- Global Health Estimates: Life expectancy and leading causes of death and disability 2019 (WHO). https://www.who.int/data/gho/data/themes/mortality-and-global-health-estimates
- Wallace LS. A View of Health Care Around the World. Ann Fam Med, 2013;11(1):84.
- What is global health? https://www.ncbi.nlm.nih.gov/pmc/articles/PMC2852240/

- Farmer P, Kim JY, Kleinman A, & Basilico M. Reimagining Global Health: An Introduction. 1st ed., University of California Press, 2013. JSTOR. http://www.jstor.org/stable/10.1525/j.ctt46n4b2. Chapters 1 and 12

Required preparation – *Be prepared to discuss the following*

- Based on the readings, how do you now define global health? Compare and contrast the terms: global health, international health, and public health?
- What is the difference between mortality, DALY and QALY? What components are used to calculate these?
- Review key findings from GBD 2019. What stands out to you? What are some possible factors that account for recent trends? How might COVID-19 impact some of this data?
- How might a focus on 'Global Health Delivery' change the approach to addressing problems in global health? Give examples of major public health efforts that you know of which take a health delivery or biosocial approach to reducing disparities in global health.
- Consider this statement: "Many failures in global health can be attributed to a lack of historical reflection and biosocial analysis." Why is this the case? What examples can you cite?
- Please review this interactive map to better understand the Global Burden of Disease and the specific impact certain diagnoses have on DALYS, based on gender, age, and country of origin. http://www.healthdata.org/data-visualization/gbd-compare. Also recall the Key Global Health Estimates on Morbidity and Mortality above. Is there a particular statistic or observation that strikes you that falls within your profession/area of training? Explore that statistic by finding a GBD article that falls within your area of interest and be prepared to discuss it as a group.
- Activity:
    - Follow this link: https://vizhub.healthdata.org/gbd-compare/
    - Click the black and white world map icon on the left side of the screen
    - Within the dropdown menu labeled 'Cause', select a disease process that falls within your field or interests. Notice the geographic differences in DALYs for your selected disease process.
    - Now click the Line Graph icon on the left side of the screen. Notice how DALYs change over time.
    - Within the 'Locations' Setting you will notice two dropdown boxes. In the lower box, click the small "x" to deselect the auto-populated countries. You'll notice the graph empties. Now within the 'Switch location group' dropdown menu, Select the emboldened "World Bank Income Levels". Notice how DALYs vary across economic groups. What observations do you make?
    - Now adjust the 'Measure' Setting to "Deaths" and then to "YLD" (Years lived with disability). What observations do you make?
    - In the group session on August 12, we will break out into small groups to share some of our findings together, so bookmark these settings. Be prepared to share your screen with your small group, and review one or two interesting observations you made during this exercise. To bookmark your graphic settings within the GBD-Compare tool, click on the 'Share' tab on the top right of the screen. Either copy or email yourself one or two interesting slides you created during this exercise to share when we meet as a group.

Discussion

Leaders

- Merranda Holmes, MD - Internal Medicine & Pediatrics
- Michael Dewan, MD, MSCI - Pediatric Neurosurgery

Discussion questions

- Choose one disease entity, injury, or disability that is relevant to your field of medicine and consider the fundamental reasons why - for that specific entity - there exists differences in given places. Please offer two interventions, changes, upgrades etc. that you think could feasibly be implemented on a large scale to address such differences.
  Here's an example... Entity: Mortality from road traffic accidents between Kampala Uganda and Nashville TN. 1) Enhance, implement, and enforce road traffic laws including helmet use for drivers and passengers on motorbikes, designated sidewalks or shoulders along high-speed thoroughfares, and speed governors on vehicles transporting more than 12 passengers. 2) State-sponsored (no charge to patient or family) neuro imaging (CT scan) for all TBI patients presenting to a hospital with a poor or declining GCS score.

Additional resources

- The Institute for Health Metrics and Evaluation. http://www.healthdata.org
- GBD and DALY Visualization. http://www.healthdata.org/data-visualization/gbd-compare
- The *Lancet* GBD Resource Centre. https://www.thelancet.com/gbd
- [For Students with a particular interest in Surgery] Global Surgery and the burden of surgical disease - Lancet Commission 2015. https://pubmed.ncbi.nlm.nih.gov/25924834/
- From MDGs to SDGs - Health in 2015. WHO. Chapter 1: General Introduction (pp 1-11) https://www.who.int/docs/default-source/gho-documents/health-in-2015-mdgs-to-sdgs/health-in-2015-from-mdgs-to-sdgs.pdf?sfvrsn=8ba61059_2
- The Sustainable Development Goals https://sdgs.un.org/2030agenda
- GBD Concepts (Breaking down DALYs) https://www.who.int/quantifying_ehimpacts/publications/en/9241546204chap3.pdf
- The art of medicine: The danger of stories in global health. Lancet. Mar 2020. https://www.thelancet.com/action/showPdf?pii=S0140-6736%2820%2930427-X

MODULE 2. SOCIOCULTURAL AWARENESS IN GLOBAL HEALTH ENGAGEMENTS

Learning Objectives

- Compare the aims of key stakeholders in global health experiences with a focus on areas of alignment and conflict.
- Reflect on ethically-, clinically-, and educationally-appropriate objectives for short-term experiences in low-resource settings based on needs of all involved.

Milestones

CUGH 10a

ACGME Milestones

- Professionalism

- Systems-based practice

Pre-reading and viewing list

- Kung TH, Richardson ET, Mabud TS, Heaney CA, Jones E, Evert J. Host community perspectives on trainees participating in short-term experiences in global health. Med Educ. 2016 Nov;50(11):1122-30.
- Eichbaum QG, Adams LV, Evert J, Ho MJ, Semali IA, van Schalkwyk SC. Decolonizing global health education: rethinking institutional partnerships and approaches. Acad Med. 2021 Mar 1;96(3):329-335.
- Brocher Declaration. Advocacy for Global Health Partnerships. https://www.ghpartnerships.org/brocher

Required preparation – *Be prepared to discuss the following*

- What are the risks of professionals and trainees participating in short-term global health experiences? What are the risks to hosting institutions and communities?
- How do short-term experiences in global health by professionals from high-resource settings benefit participants? How do they benefit host institutions and communities?
- What does "mutually beneficial" partnership for institutions engaged in global health education, research and care delivery look like?

Discussion

Leaders

- Elizabeth Rose

Discussion questions

- How might social and cultural differences contribute to misunderstanding among stakeholders? Examples may include end of life care, educational hierarchies, power and privilege differences, communication challenges.
- How might the values of equity, beneficence, and mutual respect be utilized to find agreement about selecting primary objectives for the experience?

Session Outline

This discussion will take learners through a series of vignettes to build appreciation for the perspectives and priorities of various actors involved in short-term global health experiences. If possible, have learners divide into breakout groups to discuss them separately and report their key discussion points to the group.

Video (https://youtu.be/eKFjWR7X5dU) and large group discussion (10-15 minutes)

Breakout groups: 10 minutes

Each group should discuss their vignette and the following questions

Group 1

Vignette: You are director of a new international rotation for residents. You will be supervising two residents at a clinical site you have visited before in a district-level government hospital with one local specialist physician in a country in sub-Saharan Africa. The site knows you will have trainees with you but is also eager for you assist with education for their medical training program.

- What are your primary objectives for yourself and the residents who will be participating in the rotation? Please list several objectives using any format you would like.
- If this is the first time your institution has sent residents abroad for a rotation, what are your primary concerns going into the experience? What steps can you take to connect prior to arrival and to prepare yourself and your residents?

Group 2

Vignette: You are the only specialist physician at a busy district-level hospital in a country in sub-Saharan Africa. Because of the severe shortage of trained providers in your area, you recently started a mid-level medical training program. Through a connection with your national society, you were contacted by a physician from the US who visited once, desires to volunteer at your institution and is willing to teach sub-specialty lectures to your students. You have also been notified that advanced trainees will be coming.

- What are your primary objectives for the time the volunteer physician and trainees will be working with you? Please list several objectives using any format you would like.
- If this is your first time hosting international visitors for this length of time, what are your primary concerns going into the experience?

Group 3

Vignette: You are the chair of an academic department in the US and have seen other departments pursue opportunities for their faculty and residents to participate in global health. You have also heard from your residency program director that applicants to the program are increasingly asking about international service and learning opportunities. You wonder if it may be time to establish a program in the department to begin sending faculty and trainees to underserved areas for short-term service and education opportunities.

- What are your primary objectives for establishing a global health program within your department? How might you convince the other departmental leadership that this is an important use of resources?

Group 4

Vignette: You are a mother of a child with a cleft lip living in a rural part of a country in sub-Saharan Africa. Although the local surgeon is qualified to perform the operation, she and the hospital's anesthesiologist have referred you to the capital because your child has a significant heart murmur and appears growth restricted. Unfortunately, the expense of travel and lost income required to go to the capital is too much. You have heard, however, that a visiting specialist will be coming from the US and may be willing to work with the surgeon to do the operation.

- As a member of the local community what are your goals for the health system? How might a visiting specialist impact your interaction with the local hospital and its providers?

Report Back: 20-25 minutes

Once each group has presented, the following discussion questions will be asked:

- How do short-term global health experiences (in which professionals from high-resource settings go to low-resourced settings) benefit participants? How do they benefit host institutions and communities?
- What does a "mutually beneficial" partnership for institutions engaged in global health education, research, and care delivery look like?
- In what ways might the goals of sending organizations, volunteers, hosting organizations, and communities aligned? In what ways might they be in conflict?

- How might social and cultural differences contribute to misunderstanding among stakeholders? Examples may include end of life care, educational hierarchies, power and privilege differences, communication challenges.
- How might the values of equity, beneficence, and mutual respect be utilized to find agreement about selecting primary objectives for the experience?

Additional resources

- Farmer P, Kim JY, Kleinman A, & Basilico M. Reimagining Global Health: An Introduction. 1st ed., University of California Press, 2013. JSTOR. http://www.jstor.org/stable/10.1525/j.ctt46n4b2. Chapter 4: Health for All? Competing Theories and Geopolitics.
- Rose E. Traveling Abroad and Culture Shock. VIGH Global Health Education Videos.
- Crump JA, Sugarman J. Ethical considerations for short-term experiences by trainees in global health. JAMA 2008;300(12):1456-8

MODULE 3. CAPACITY STRENGTHENING

Learning Objectives

- Collaborate with a host or partner organization to assess the organization's operational capacity.
- Co-create strategies with the community to strengthen community capabilities and contribute to reduction in health disparities and improvement of community health.
- Integrate community assets and resources to improve the health of individuals and populations.

Milestones

CUGH Global Health Competencies 4a, 4b, 4c

ACGME Milestones

- Systems-based practice
- Practice-based learning and improvement

Pre-reading and viewing list

- Harges D. Making the invisible visible [TEDx Indianapolis]. 2014, Nov 18. https://www.youtube.com/watch?v=y6yiRXVP90g
- Farmer P, Kim JY, Kleinman A, & Basilico M. Reimagining Global Health: An Introduction. 1st ed., University of California Press, 2013. JSTOR. http://www.jstor.org/stable/10.1525/j.ctt46n4b2. Chapter 6: Building an effective Rural Health Delivery Model in Haiti and Rwanda *(read pages 135-141, 167-172 and 179-182)* and Chapter 7: Scaling Up Health Delivery Models Worldwide.
- Crisp BR, Swerissen H, Duckett SJ. Four approaches to capacity building in health: consequences for measurement and accountability. Health Promot Int. 2000;15(2):99-107.
- Russell C & Smeaton T. From needs to assets: Charting a sustainable path towards development in sub-Saharan African countries. 2009. United Kingdom: Practical Action Publishing. *(read pages 1-6)*

- Setting SMART goals, A Quick Overview https://www.youtube.com/watch?v=1-SvuFIQjK8&t=29

Required preparation – *Be prepared to discuss the following*

- How critical is the role of the Public Sector/Government in capacity strengthening?
- What are the factors influencing Human Capital Flight ("Brain Drain" and "Brain Gain") What are the net costs and net benefits for the migrants and countries involved? What are some of the possible solutions to Human Capital Flight?

Bonus Questions

- Based on the readings, how do you define capacity strengthening? What are some of the core indicators or measures of capacity strengthening? How do we evaluate the outcomes or impacts of capacity strengthening efforts?
- How would you apply concepts from readings to how you might form your own partnerships with organizations or communities?

Discussion

Leaders

- Joseline Haizel-Cobbina, MBChB, MPH
- Chris Bonfield, MD

Discussion questions

- Reflect on this statement: "External assistance/provision is to increase the self-sustaining ability of people to recognize, analyze and solve their problems by more effectively controlling and using their own and external resources and not for the external provider to control projects it has resourced."
- What are the power dynamics at play in capacity strengthening partnerships? Discuss how power dynamics may positively or negatively impact capacity strengthening efforts.

Session Outline

Case Vignette

Haiti has been grappling with inadequate healthcare infrastructure over the last couple of decades. According to the Ministry of Health, even before the 2010 earthquake, Haiti's healthcare system was not able to respond to the needs for basic healthcare services. The earthquake worsened the situation, destroying 50 healthcare centers as well as the Ministry of Health building. The current political conflict is further putting a strain on access to healthcare in Haiti. You are part of a team of Global Health experts visiting Haiti to support the work of Haiti's Ministry of Health to rebuild their health infrastructure:

1. What are the steps you will take to create and maintain a partnership with the Ministry of Health?
2. What are some of measures/indicators needed to assess the operational capacity of Haiti's health system?
3. Identify some health challenges currently being faced by Haiti and create SMART goals for each health challenge identified.
4. Develop strategies to achieve the SMART goals listed above.
5. Which community development approach/strategy will you use aimed at improving the health of Haitians? Asset Based or need based, and why? How do you intend to do this?

Additional resources

- Wallerstein N, Muhammad M, Sanchez-Youngman S, et al. Power Dynamics in Community-Based Participatory Research: A Multiple–Case Study Analysis of Partnering Contexts, Histories, and Practices. Health Educ Behav. 2019;46(1_suppl):19S-32S.
- World Health Organization, ed. Monitoring the Building Blocks of Health Systems: A Handbook of Indicators and Their Measurement Strategies. World Health Organization; 2010.
- Duncan DH. The classic duo: Accountability and community development can help unlock an abundance of resources. Public Management Magazine. 2012; 20–23.
- Loh LC, Cherniak W, Dreifuss BA, Dacso MM, Lin HC, Evert J. Short term global health experiences and local partnership models: a framework. Glob Health. 2015;11(1):50.

MODULE 4. SOCIAL CONSIDERATIONS OF HEALTH AND PLANETARY HEALTH

Learning Objectives

- Describe how cultural context influences perceptions of health and disease.
- List major social and economic effects on health and their impacts on the access to and quality of health services and on differences in morbidity and mortality between and within countries.
- Understanding the interconnectedness of human and environmental health
- Describe how human activities are altering natural systems like climate, biodiversity, and biogeochemical cycles.
- Explain how these changes affect human health, including the exacerbation of existing conditions, emergence of new diseases, impacts on mental health, and the global distribution of these effects.
- Explain the role of health systems in promoting environmental sustainability and identify strategies for creating climate-resilient health systems

Milestones

CUGH Global Health Competencies 3a, 3b, 3c

ACGME Milestones

- Systems-based practice
- Practice-based learning and improvement
- Medical knowledge

Pre-reading and viewing list

- Manchanda, R. What makes us get sick? A look upstream. TEDTalk 2014.
- Prüss-Üstün A, Corvalán C. Preventing disease through healthy environments: Towards an estimate of the environmental burden of disease: Executive summary. 2006. Geneva, Switzerland: World Health Organization.
- Introduction to Planetary Health; Planetary Health Alliance: https://youtu.be/_b2yVNkPgvM
- Myers SS. Planetary health: protecting human health on a rapidly changing planet. *Lancet*. 2017;390(10114):2860-2868.
    - **Required preparation** – *Be prepared to discuss the following*

- Describe a sociocultural approach to health.
- Choose an example of a communicable or non-communicable disease and describe biosocial factors responsible for their prevalence in low/middle income countries.
- Describe two cultural practices that promote health. Describe two cultural practices that are harmful.
- List three different ways in which planetary health is changing over time and how these changes may affect human health and the health of the planet. Consider how these effects may affect people and communities around the world differently.

Discussion

Leader

- Lindsey Zamora

Additional resources

- Woolf SH & Aron L (Eds.). US health in international perspective: Shorter lives, poorer health. 2013. Washington, DC: National Academies Press. https://pubmed.ncbi.nlm.nih.gov/24006554/
- United Nations. Sustainable Development Goals. https://www.un.org/sustainabledevelopment/sustainable-development-goals/
- Planetary Health is Public Health; APHA and Planetary Health Alliance; https://www.youtube.com/watch?v=D6YOjJcSvA0
- The Nature Conservancy; Climate Strong Communities: https://www.nature.org/en-us/get-involved/how-to-help/climate-strong-communities/?en_txn1=p_g.gd.eg.ec_csc.CSC.LSG&gad_source=2&gad_campaignid=22612830221&gclid=CjwKCAjw6s7CBhACEiwAuHQcksgLrXC222Xp4N7U1RkHl25vaK2b9xThuyuuVLTmRP8NITU_6DUQMBoC8FIQAvD_BwE&gclsrc=aw.ds
- Planetary Health; Protecting Global Health on a Rapidly Changing Planet, Grand Rounds Webinar Massachusetts General Hospital: https://www.youtube.com/watch?v=8fYSpSXW9N8
- EAT Lancet Commission on Food, Planet, and Health; Planetary Health Diet: https://eatforum.org/eat-lancet-commission/the-planetary-health-diet-and-you/
- Planetary Health Alliance: https://planetaryhealthalliance.org/what-is-planetary-health/

MODULE 5. GLOBALIZATION OF HEALTH AND HEALTHCARE

Learning Objectives

- Describe different national models or health systems for provision of healthcare and their respective effects on health and healthcare expenditure.
- Describe how global trends in healthcare practice, commerce and culture, multinational agreements and multinational organizations contribute to the quality and availability of health and healthcare locally and internationally.
- Describe general trends and influences in the global availability and movement of health care workers.

Milestones

CUGH Global Health Competencies 2a, 2b, 2d

ACGME Milestones

- Systems-based practice
- Practice-based learning and improvement

Pre-reading and viewing list

- Redditt V, ole-Moi Yoi K, Rodriguez W, Talbot JR, Weintraub R. Malaria Control in Zambia (Condensed Version). Harvard Business Publishing. 2012.
- World Health Organization & Alliance for Health Policy and Systems Research. Systems thinking for health systems strengthening. (2010, May 14). [Video file - 6:30]
- Global Health with Greg Martin. Health Systems. [Video file - 7:15]
- Martin MH. Global Health Systems, Governance and Actors. VIGH Global Health Education Videos. [Video file - 10:00]
- WHO. Global strategy on human resources for health: Workforce 2030. *Summary and Background*. Geneva. 2015;7-14.

Required preparation – *Be prepared to discuss the following*

- Describe the building blocks of a healthcare ecosystem. How does considering the interrelatedness of these components influence efforts to provide health systems strengthening? What are examples?
- What are the key elements of a high-quality health system? Do these elements differ between health systems in high-, middle- and low-income countries?
- Using the WHO Workforce 2030 document, describe general trends and influences on the global availability and movement of healthcare workers. Choose one objective from Workforce 2030 and summarize a policy option proposed for LMICs to advance toward stated milestones.
- Identify various stakeholders in global healthcare market. What are their interests and how might they affect operations at the country level?
- Using the case study 'Malaria Control in Zambia', map out the healthcare ecosystem around malaria prevention, treatment and management in Zambia from the individual to the national level. Who are the key players? What were the key building blocks for a comprehensive response? Why was this strategic plan effective?

Discussion

Leader

- Matt Kynes

Discussion questions

During this month's session we focused on globalization and the healthcare ecosystem.

- What are the benefits of globalization to development of healthcare systems in low-resource settings?
- What are the harms of globalization in these settings? Discuss how the involvement of an external actor (such as a funding agency) in a healthcare ecosystem can have unforeseen helpful or harmful consequences in other parts of the system?

Additional resources

- De Savigny D, Taghreed A (eds). Systems thinking for health system strengthening. Full report. World Health Organization & Alliance for Health Policy and Systems Research. (2009). https://www.who.int/alliance-hpsr/resources/9789241563895/en/
- Bloom DE, Khoury A, Subbaraman R. The promise and peril of universal health care. *Science*. 2018;361(6404):eaat9644.
- Kaiser Family Foundation. Global Health: A Primer. https://www.kff.org/report-section/the-u-s-government-engagement-in-global-health-a-primer-report/
- Frenk J, Gómez-Dantés O, Moon S. From sovereignty to solidarity: a renewed concept of global health for an era of complex interdependence. Lancet. 2014;383(9911):94-97.
- McCoy D, Chand S, Sridhar D. Global health funding: how much, where it comes from and where it goes. Health policy and planning. 2009 Nov 1;24(6):407-17.
- Kim JY, Farmer P, Porter ME. Redefining global healthcare delivery. Lancet 2013; 382(9897):1060-9.

MODULE 6. IMMIGRANT AND REFUGEE HEALTH

Learning Objectives

- Understand the unique challenges facing immigrant populations worldwide in obtaining and navigating healthcare.
- Apply justice and human rights principles in addressing global health problems.

Milestones

CUGH Global Health Competencies: 8a, 8c, 8e, 8f, 11a, 11c

ACGME Milestones

- Patient care
- Medical knowledge
- Professionalism
- Practice-based learning and improvement
- Systems-based practice

Pre-reading and viewing list

- Backman G, Hunt P, Khosla R, Jaramillo-Strouss C, Fikre BM, Rumble C, Pevalin D, Páez DA, Pineda MA, Frisancho A, Tarco D. Health systems and the right to health: an assessment of 194 countries. Lancet. 2008 Dec 13;372(9655):2047-85.
- Capps R, Newland K, Fratzke S, Groves S, Auclair G, Fix M, McHugh M. Integrating refugees in the United States: The successes and challenges of resettlement in a global context. Statistical Journal of the IAOS. 2015 Jan 1;31(3):341-67. (read the executive summary on pages 1-2 and then scan the rest of the document)
- I-PACK Immigration and Asylum PowerPoint from sugarprep.org

Required preparation – *Be prepared to discuss the following*

- Identify a justice issue that faces your community and propose a strategy to enhance address this issue.

- Identify an ethnic group in your community. What health disparities exist in this group? Reflect on what you learn about that ethnic group in regard to their access to health services, including mental health services. What language barriers exist? What biases did you observe from others or your own? Think about what you learned and how you would apply this in a global health setting.

Discussion

Leaders

- Lindsey Zamora
- Siloam Healthcare

Discussion questions

Think about a patient or patient group you've worked with in the past that would be classified as either an economic migrant, refugee, or asylum seeker.

- What types of unique healthcare concerns did this patient or patients have?
- How could you tailor your care to meet the specific needs of the patient(s)?

Additional resources

- United Nations. Universal Declaration of Human Rights. 1948. Paris: United Nations.
- “Your path to our health” video series on the right of the page: https://www.rwjf.org/en/library/research/2017/09/immigration-status-and-health.html
- World Health Organization. Health and Human Rights. 2015. Fact Sheet Number 323.
- Ahn R, Alpert EJ, Purcell G, Konstantopoulos WM, McGahan A, Cafferty E, Eckardt M, Conn KL, Cappetta K, Burke TF. Human trafficking: review of educational resources for health professionals. Am J of Preventive Medicine. 2013 Mar 1;44(3):283-9.
- UNHCR, The United Nations High Commissioner for Refugees. http://www.unhcr.org/cgi-bin/texis/vtx/home
- Physicians for Human Rights. http://physiciansforhumanrights.org/?referrer=https://www.google.com/
- Amnesty International. http://www.amnestyusa.org

MODULE 7. COLLABORATION, PARTNERING AND COMMUNICATION

Learning Objectives

- Understand what it means to collaborate/partner with diverse stakeholders to establish a successful academic partnership.
- Identify key concepts of successful academic partnerships.
- Discuss why building trust with local community members/stakeholders is the cornerstone to successful projects, research and programs.

Milestones

CUGH Global Health Competencies 5a, 5b, 5f

ACGME Milestones

- Systems-based practice

- Professionalism
- Interpersonal and Communication Skills

Pre-reading and viewing list

- John CC, Ayodo G, Musoke P. Successful global health research partnerships: what makes them work?. AJTMH. 2016 Jan 1;94(1):5.
- Christopher S, Watts V, McCormick AK, Young S. Building and maintaining trust in a community-based participatory research partnership. Am J of Public Health. 2008 Aug;98(8):1398-406.
- Yates BA. Luke 9:2 Ministries: Safe Water Project. SharePoint Link
- Loewenberg S. Learning from failure. New York Times. 2013 February 1.
- Forget NP, Rohde JP, Rambaran N, Rambaran M, Wright SW. Emergency medicine in Guyana: lessons from developing the country's first degree-conferring residency program. Western Journal of Emergency Medicine. 2013 Sep;14(5):477.
- Busse H, Aboneh EA, Tefera G. Learning from developing countries in strengthening health systems: an evaluation of personal and professional impact among global health volunteers at Addis Ababa University's Tikur Anbessa Specialized Hospital (Ethiopia). Globalization and Health. 2014 Dec;10:1-7.

Required preparation – *Be prepared to discuss the following*

- What does it mean to establish academic partnerships?
- What are key components in establishing and maintaining successful academic partnerships?
- What does community-based participatory research project mean? Discuss several tactics for building and maintaining trust with community from a research project standpoint?
- Be prepared to use the knowledge gained from your required reading to participate in a panel discussion about projects that did and did not involve key community stakeholders and how this led to the success and/or failure of projects.

Discussion

Leaders

- Kristen Dettorre
- Panel discussion participants: BethAnn Yakes (Vanderbilt, Luke 9:2 Nonprofit); Guyanese Emergency Medicine colleagues

Discussion questions

- Please take a few moments to think about what makes a partnership successful. How do you measure this success?
- What are some methods of objectively measuring the success/failure of a partnership?

Additional resources

- Afsana K, Habte D, Hatfield J, Murphy J, Neufeld V. Partnership assessment toolkit. Ottawa: Canadian Coalition for Global Health Research. 2009 Dec.
- Crump JA, Sugarman J, Working Group on Ethics Guidelines for Global Health Training (WEIGHT). Ethics and best practice guidelines for training experiences in global health. AJTMH. 2010 Dec 12;83(6):1178.

- Damberger D. What happens when an NGO admits failures [TEDxYYC]. 2011, April 21.
- Giachello AL (author); Ashton D, Kyler P, Rodriguez ES, Shanker R, Umemoto A, (eds.) Making Community Partnerships Work: A Toolkit. 2007. White Plains, NY: March of Dimes Foundation.
- Gormley G, Guyer-Miller L, & Training Resources Group. Partnership building: Practical tools to help you create, strengthen, assess, and manage your partnership or alliance more productivity. 2007. Chapel Hill, NC: The Capacity Project.
- Riviello R, Ozgediz D, Hsia RY, Azzie G, Newton M, Tarpley J. Role of collaborative academic partnerships in surgical training, education, and provision. World J Surg. 2010;34(3):459-465.

MODULE 8. INFECTIONS AND EFFECTS

Learning Objectives

- Describe what is meant by the term "structural violence" in the context of the global burden of disease
- Elucidate the relationship between poverty and disease, including specific past and present examples
- Describe ways to incorporate knowledge of the link between poverty and disease into your practice
- Describe how travel and trade contribute to the spread of communicable and chronic diseases.
- Describe the relationship between access to and quality of water, sanitation, food and air on individual and population health.
- Articulate barriers to health and healthcare in low-resource settings locally and internationally.
- Demonstrate the ability to adapt clinical or discipline-specific skills and practice in a resource-constrained setting.
- Identify how demographic and other major factors can influence patterns of morbidity, mortality, and disability in a defined population.

Milestones

CUGH Global Health Competencies: 7a, 7b, 7c

ACGME Milestones

- Systems-based practice
- Medical knowledge

Pre-reading and viewing list

- *priority viewing: A Reflection of Growing Inequality: Dr. Paul Farmer on the Deadly Ebola Outbreak in West Africa [Video file – 11:00]
- Farmer PE. Shattuck Lecture. Chronic infectious disease and the future of health care delivery. N Engl J Med. 2013 Dec 19;369(25):2424-36. (If you prefer, feel free to read Dr. Farmer's book, *Infections and Inequalities: The Modern Plagues*)

- Dybul M. A social movement to fight AIDS, malaria, and TB and end the epidemics. TEDxAmRing. [Video file – 16:00]
- Pacete J. How pandemics spread: Introduction to infectious diseases. TEDEd 2014. [Video file – 8:00]
- Manchanda R. What makes us get sick? (Look upstream). TEDTalks 2015. [Video file – 18:22].

Required preparation – *Be prepared to discuss the following*

- What are the main factors that determine your personal health? What are the main factors that would determine the health of a poor person in a poor country?
- If you could only pick one indicator to describe the health status of a poor country, which indicator would you use and why?
- Summarize how travel and trade contribute to the spread of communicable and chronic diseases.
- List three different diseases or conditions that can be caused by each of the following: poor water quality; poor sanitation; lack of access to safe, healthy food; and poor air quality.
- How do barriers to care differ by specialty or service (for example, access to primary care versus to mental healthcare versus to subspecialty care)?

Discussion

Leader

- Jon Niconchuk

Discussion question

- Think about a patient you have seen whose disease process or care received was directly impacted by something wholly out of their own control. What is the role of the physician in society when public policies or societal- constructs (aka "structural violence") affect your patient's health?

Additional resources

- CUGH. Parasitic Infectious Diseases. https://www.cugh.org/resources/educational-products/infectious-parasitic-communicable-diseases/
- Farmer PE. *Infections and Inequalities: The Modern Plagues*. 1st ed., University of California Press, 1999.

MODULE 9. SURGERY, ANESTHESIA AND OBSTETRICS

Learning Objectives

- Describe general trends and influences in the global availability and movement of health care workers.
- Collaborate with a host or partner organization to assess the organization’s operational capacity.
- Co-create strategies with the community to strengthen community capabilities and contribute to reduction in health disparities and improvement of community health.

- Include representatives of diverse constituencies in community partnerships and foster interactive learning with these partners.
- Demonstrate diplomacy and build trust with community partners.
- Acknowledge one's limitations in skills, knowledge, and abilities.
- Articulate barriers to health and healthcare in low-resource settings locally and internationally.
- Demonstrate the ability to adapt clinical or discipline-specific skills and practice in a resource-constrained setting.
- Identify how demographic and other major factors can influence patterns of morbidity, mortality, and disability in a defined population.
- Conduct a situational analysis across a range of cultural, economic, and health contexts.

Milestones

CUGH Global Health Competencies 7a, 7b, 7c

Pre-reading and viewing list

- Say L, Chou D, Gemmill A, et al. Global causes of maternal death: a WHO systematic analysis. Lancet Glob Health 2014; 2:e323-33
- TA Reynolds. The impact of trauma care systems in low-and-middle income countries. Annu Rev Public Health. 2017 Mar 20:38;507-532.
- Hunter DJ, Reddy KS. Noncommunicable diseases. N Engl J Med. 2013;369(14):1336-1343.
- World Health Organization. Saving lives, spending less: a strategic response to noncommunicable diseases. World Health Organization; 2018.
- Disease Control Priorities, Vol. 3 – Essential Surgery. Chapter 15: Anesthesia and Perioperative Care http://dcp-3.org/chapter/1796/anesthesia-and-perioperative-care
- Law TJ, Lipnick M, Joshi M, Rath GP, Gelb AW. The path to safe and accessible anaesthesia care. The path to safe and accessible anaesthesia care. Indian J Anaesth 2019;63:965-71.
- McQueen K. Solving the Global Anesthesia Crisis. TEDx Nashville. 2017 Sept. [Video file - 15:00]
- Meara J et al. Global Surgery 2030: Evidence and solutions for achieving health, welfare and economic development. Int J Obstet Anesth 2016 Feb;25:75-8.
- Farmer PE. Surgery and global health: a view from beyond the OR. World J Surg 2008 Apr;32(4):533-6.
- Why did Mrs. X die? [Video file – 14:37]

Discussion

Leader

- Ryan Belcher

Discussion questions

- If the government of a low- and middle-income country is not able or willing to provide the resources for health infrastructure to sustain/improve the surgery/anesthesia/obstetrics divisions, how can these fields improve or provide the care their population needs?
- How can "brain drain" be prevented?

MODULE 10. PROGRAM MANAGEMENT

Learning Objectives

By the end of this module, participants will be able to:

- Understand and use various tools for program development, management and evaluation including the Theory of Change (TOC), SMART objectives, logical frameworks, stakeholder mapping, Gantt Charts, and budgets.
- Apply principles of program management to a global health issue or intervention.
- Plan, implement and evaluate an evidence-based program.
- Apply project management techniques throughout program planning, implementation, and evaluation

Milestones

CUGH Competencies 9a, 9b

ACGME Milestones

- Problem-based learning and improvement
- Systems based practice
- Communication and interpersonal skills
- Professionalism

Pre-reading and viewing list

Videos

- Martin MH. Project Management Overview (Part 1). VIGH Global Health Education Videos. [Video file - 16:05]
- Martin MH. Project Management Tools (Part 2). VIGH Global Health Education Videos. [Video file - 21:54]
- *Supplementary videos:* Martin MH. Global health funding. VIGH Global Health Education Videos. [Video file - 10:00]

Articles

- Centers for Disease Control and Prevention (CDC). Writing SMART objectives. 2009; Evaluation briefs, 3B, 1–2.
- W. K. Kellogg Foundation. Logic Model Introduction.
- Hailemariam M, Fekadu A, Selamu M, Alem A, Medhin G, Giorgis TW, DeSilva M, Breuer E. Developing a mental health care plan in a low resource setting: the theory of change approach. BMC health services research. 2015 Jun;15:1-1.
- Loh LC, Cherniak W, Dreifuss BA, Dacso MM, Lin HC, Evert J. Short term global health experiences and local partnership models: a framework. Globalization and Health. 2015 Dec;11:1-7.
- O'Connor-Fleming ML, Parker E, Higgins H, Gould T. A framework for evaluating health promotion programs. Health Promotion Journal of Australia. 2006;17(1):61-6.

Required preparation – *Be prepared to discuss the following*

CUGH Competency Questions:

- What are the key principles to consider when planning a community-based or global health program?
- Propose one SMART objective for evaluating a global health program.
- In your own words, describe a model for planning and assessing the outcomes of a community-based program.
- Present the specific steps involved in program planning, implementation and evaluation for a Case Study-based global health issue.
- Who needs to be involved at various stages?
- What organizations will you look to for help at each stage?
- How do you involve all stakeholders?
- How would you engage the community in program planning, implementation, and evaluation?
- In what ways can the development of global programs help and hurt existing national health systems?

Discussion

Leaders

- Marie Martin
- Guest: Reeta Gobin, Dean of the College of Medicine, University of Guyana

Discussion questions

- What are some key principles to consider when planning a global health program?
- How would you use one or more program management tools in a future global health project? If you already use one of these tools, how might you adapt or enhance it after the videos and readings for this session? Are there considerations that should be kept in mind when using program management tools? What are some possible risks or limitations?

Additional resources

- Program development
  - Unite for Sight Global Health University. Certificate in Effective Program Development. http://www.uniteforsight.org/effective-program-development/certificate.
- Logic model frameworks
  - Serowoky ML, George N, Yarandi H. Using the program logic model to evaluate¡ Cuídate!: A sexual health program for Latino adolescents in a school-based health center. Worldviews on Evidence-Based Nursing. 2015 Oct;12(5):297-305. https://www.ncbi.nlm.nih.gov/pubmed/26422189
  - Kellogg Foundation logic model development guide https://www.wkkf.org/resource-directory/resources/2004/01/logic-model-development-guide
- Stakeholder mapping
  - Davis K. An empirical investigation into different stakeholder groups perception of project success. International Journal of Project Management. 2017 May 1;35(4):604-17.
- Program Evaluation

- International Consortium for Health Outcomes Measurement. http://www.ichom.org/measure
- International Initiative for Impact Evaluation. http://www.3ieimpact.org/en/publications
- U.S. Department of Health and Human Services, & Centers for Disease Control and Prevention. Introduction to program evaluation for public health programs: A self-study guide. 2011. Atlanta, GA: Centers for Disease Control and Prevention. http://www.cdc.gov/eval/guide/CDCEvalManual.pdf

MODULE 11. ETHICS AND NEOCOLONIALISM

Learning Objectives

- Demonstrate an understanding of and an ability to resolve common ethical issues and challenges that arise when working in low-resource settings to address global health issues
- Demonstrate an awareness of local and national codes of ethics relevant to one's working environment
- Apply the fundamental principles of international standards for the protection of human subjects in diverse cultural settings
- Dismantle and re-imagine global health practice and research to be mutually beneficial
- Explain how the history of colonization impacts global health ethics and the field of global health

Milestones

CUGH Global Health Competencies: 6a, 6b, 6c

ACGME Milestones

- Patient care
- Professionalism
- Medical knowledge

Pre-reading and viewing list

- Crump JS, Sugarman J, & WEIGHT. Ethics and best practice guidelines for training experiences in global health. AJTMH. 2010;83(6):1178-1182.
- Murphy J, Hatfield J, Afsana K, Neufeld V. Making a commitment to ethics in global health research partnerships: A practical tool to support ethical practice. Journal of Bioethical Inequality. 2015;12(1):137-146.
- Ethical Challenges in Short-Term Global Health Cases www.Ethicsandglobalhealth.org
- Rose ES. Professionalism and ethics in global health experiences. VIGH Global Health Education Videos. [Video file -18:41]

Required preparation – *Be prepared to discuss the following*

- What do you perceive to be the primary ethical concerns when working with healthcare professionals in an unfamiliar setting?
- How might the educational and professional expectations at your home institution differ from those on your global health elective and how will you manage those conflicts?

- What ethical considerations accompany global health partnerships, whether they be clinical, educational, or research partnerships? What knowledge, attitudes and skills are needed to ensure that these partnerships are mutually beneficial and ethically responsive to home and host institutions?
- Describe considerations important to the process of informed consent when working in a resource-limited or cross-cultural setting.

Discussion

Leader

- Rondi Kauffmann

Discussion questions

- Describe an ethical dilemma you have witnessed or experienced during your global health work. How did you respond? What did you learn? What changes have you made to your practice as a result of that experience?
- You are on a global health elective rotation in another country. You are scheduled to scrub in the OR with a host faculty member and several residents. When you enter the OR, you notice a resident wearing a sterile gown with a hole in the front, revealing unsterile clothing beneath. The host country faculty member makes no comment as they approach the OR table. What is your initial reaction? Why would you speak up in this situation? Why might you not say something? Would additional information help you make the decision? If you choose to say something, what kinds of things could you say and whom would you say it to? How would you handle this situation at your home institution? Is it different from how you would handle it when you are visiting?

Additional resources

- Wall AE. Ethics in global surgery. World J Surg. 2014;38(7):1574-1580.
- Wall AE. Ethics for International Medicine: A Practical Guide for Aid Workers in Developing Countries (Geisel Series in Global Health and Medicine). 2012. https://www.amazon.com/Ethics-International-Medicine-Practical-Developing/dp/161168210X
- Unite for Sight Ethics Course https://www.uniteforsight.org/global-health-course/
- "First, Do No Harm" video documentary https://vimeo.com/22008886

BIBLIOGRAPHY

Additional Resources

- Health Systems and Governance
    - https://www.cugh.org/resources/educational-products/health-systems-management-governance/
- CUGH Educational Resources
    - https://www.cugh.org/resources/educational-products/

Selected Films

- A Closer Walk (film about the global AIDS pandemic)

- The Lost Boys of Sudan (film about refugees of civil war in Sudan)
- Rx for Survival (film about global health challenges)
- The Checklist Effect

Selected Books

- Infectious and Inequalities (Paul Farmer)
- Ethics in Global Surgery (Anji Wall)
- Where There is No Doctor (David Werner)
- Mountains Beyond Mountains (Tracy Kidder)

Table S2: Demographics of Respondents (pre-course survey)

Category	Yes (n, %)
Specialty (n=56)	
Anesthesiology	8, 14%
Emergency medicine	7, 13%
Geriatric medicine	1, 2%
Gastrointestinal	1, 2%
Internal medicine	10, 18%
Med-peds	5, 9%
Neurosurgery	2, 4%
Obstetrics-gynecology	2, 4%
Ophthalmology	2, 4%
Otolaryngology	2, 4%
Pediatrics	3, 5%
Surgery	13, 23%
Female (n=58)	37, 64%
Race (n=56)	
American Indian	0, 0%
Asian	8, 14%
Black	6, 11%
Native Hawaiian	0, 0%
White	43, 77%
Other	2, 4%
Hispanic or Latino (n=54)	7, 13%
Global Health experience (n=59)	
Volunteer	37, 63%
Leisure travel	34, 58%
Study abroad	24, 41%
Work abroad	23, 39%
Trained in global health	21, 36%
Faith-based trip	17, 29%
Born/raised abroad	16, 27%
Internship abroad	10, 17%
Other	4, 7%
None	3, 5%
Desired ways to incorporate Global Health into career (n=59)	
Conduct projects abroad	44, 75%

Work with foreign-born populations in the United States	38, 64%
Short-term mission trips (e.g., 1 week – 2 months)	35, 59%
Practice abroad 2-6 months per year	22, 37%
Practice abroad 6-8 months per year	4, 7%
Practice abroad 9-12 months per year	4, 7%

Table S3: Pre- and Post-course Means (and Percent Change from Pre- to Post-course) ofSelf-reported Level of Knowledge for the Course Competencies (1=very low; 5=very high)

**Criteria:**<br>**Understanding of the following concepts…**	**Mean (SD), pre-course (n=59)**	**Mean (SD), post-course (n=19)**	**Percent change, pre- to post-course**
Trends and influences in the global healthcare workforce	2.37 (0.72)	4.05 (0.78)	71%
Approaches for the specific discipline of the global health practitioner	2.39 (0.79)	4.00 (0.97)	67%
Program management in the design, implementation, and evaluation of global health programs	2.44 (0.75)	3.95 (1.03)	62%
Globalization impacts on health, health systems, and healthcare delivery	2.54 (0.73)	3.95 (0.85)	55%
Collaboration in selecting, recruiting, communicating, and working with global health teams	2.68 (0.77)	4.16 (0.83)	55%
Sociocultural and political awareness across cultural settings and international political landscapes	2.66 (0.76)	4.11 (0.74)	54%
Capacity strengthening to enhance global public health infrastructure and workforce	2.68 (0.74)	3.95 (1.08)	47%
Ethics in global health	2.86 (0.78)	4.21 (0.54)	47%
Health frameworks to address differences in health outcomes	2.85 (0.77)	4.05 (0.71)	42%
Causes of morbidity and mortality and variations among regions	2.78 (0.72)	3.89 (0.88)	40%
Social, economic, and environmental considerations of health	3.29 (0.73)	4.32 (0.67)	31%
**Overall**	**2.68 (0.77)**	**4.06 (0.83)**	**51%**

SD = standard deviation

Table S4: Self-reported Level of Confidence in Skills Post-course (1=very low; 5=very high)(n=19)

Criteria:<br>Confidence in each of the following skills…	Mean	Standard deviation
Apply basic principles of ethics to global health issues and settings	4.16	0.69
Identify social, economic, and environmental considerations of health	4.11	0.66
Identify approaches necessary global health practitioners	4.11	0.74
Apply understanding of how globalization affects health, health systems, and health care delivery	4.00	0.67
Explain the capacity strengthening as the sharing of knowledge/skills for enhancing global public health infrastructure and workforce	3.95	0.91
Utilize sociocultural and political awareness to work effectively within diverse cultural settings and across international political landscapes	3.89	0.74
Collaborate with various global health stakeholders to advance research, policy, and practice and to foster open dialogue and effective communication	3.84	0.90
Utilize health frameworks to address differences in health outcomes	3.84	0.69
Explain how program management supports the design, implementation, and evaluation of global health programs	3.83	0.99
Utilize understanding of the major causes of morbidity and mortality and their variations between regions to reduce health disparities globally	3.68	0.89
**Mean**	**3.94**	**0.15**

Table S5: Themes with exemplar quotes from residents

Theme	Exemplar quotes from residents
Connecting across specialties	"*training with residents from other specialties enriches the educational experience, enhances problem-solving skills, improves communication and teamwork, expands knowledge, and ultimately leads to improved patient care*"<br><br>*"it brought unique viewpoints and allowed me to consider aspects of medicine that I do not routinely think about*"<br><br>"*it was amazing to learn and see the way colleagues in all departments think about global health and what they are already doing in these spaces*"
Learning from guest lecturers	"*the most impactful lectures from the series were those that were presented directly from community partners*"<br><br>*"I gained perspective from individuals in a variety of global health settings and hearing how they approach, think about, and overcome global health challenges*"<br><br>"*broadened [my] perspective on global health, challenges faced by different specialties, and recognizing the multidisciplinary nature of global health care"*
